# Bearing Fault Diagnosis Using Multidomain Fusion-Based Vibration Imaging and Multitask Learning

**DOI:** 10.3390/s22010056

**Published:** 2021-12-22

**Authors:** Md Junayed Hasan, M. M. Manjurul Islam, Jong-Myon Kim

**Affiliations:** 1 Department of Electrical, Electronics and Computer Engineering, University of Ulsan, Ulsan 44610, Korea; junhasan@mail.ulsan.ac.kr; 2 Department of Computer Science, American International University-Bangladesh, Dhaka 1229, Bangladesh; manjurul@aiub.edu

**Keywords:** bearing, deep learning, fault diagnosis, multi-task learning, variable operating conditions, vibration imaging

## Abstract

Statistical features extraction from bearing fault signals requires a substantial level of knowledge and domain expertise. Furthermore, existing feature extraction techniques are mostly confined to selective feature extraction methods namely, time-domain, frequency-domain, or time-frequency domain statistical parameters. Vibration signals of bearing fault are highly non-linear and non-stationary making it cumbersome to extract relevant information for existing methodologies. This process even became more complicated when the bearing operates at variable speeds and load conditions. To address these challenges, this study develops an autonomous diagnostic system that combines signal-to-image transformation techniques for multi-domain information with convolutional neural network (CNN)-aided multitask learning (MTL). To address variable operating conditions, a composite color image is created by fusing information from multi-domains, such as the raw time-domain signal, the spectrum of the time-domain signal, and the envelope spectrum of the time-frequency analysis. This 2-D composite image, named multi-domain fusion-based vibration imaging (*MDFVI*), is highly effective in generating a unique pattern even with variable speeds and loads. Following that, these *MDFVI* images are fed to the proposed MTL-based CNN architecture to identify faults in variable speed and health conditions concurrently. The proposed method is tested on two benchmark datasets from the bearing experiment. The experimental results suggested that the proposed method outperformed state-of-the-arts in both datasets.

## 1. Introduction

Rotating machinery has become faster and more intelligent in recent years due to rapid innovation, and plays an increasingly vital role in many industries [1,2]. With this growth in popularity, maintenance procedures are necessary due to the critical nature of several vulnerabilities [3,4]. Rolling element bearings are the most critical components of rotating machinery. Severe working environments, alternative load conditions, and several other factors contribute to the failure of rolling element components of bearing which resulted in massive economic losses and fatalities [5]. Therefore, during the past few decades, industries have acknowledged the significance of establishing practical and dependable condition monitoring systems to address these concerns [6]. However, the acquired vibration signals from these bearings are non-stationary and non-linear in nature due to differences in clearance, friction, loads, and speed. Therefore, directly extracting significant feature information from those signals, or employing time and/or frequency domain-based analysis, is difficult [7]. As a result, developing a novel and effective method for monitoring the condition of bearings has become a difficult and worthwhile challenge [8,9].

Vibration technologies have become an increasingly popular approach in the development of a diagnostic framework for rolling bearings over the past few years [10,11]. Therefore, a tremendous amount of effort has been put into analyzing vibration signals to identify the health characteristics of rolling bearings. For instance, Zheng et al. [12] proposed a diagnostic feature formation technique by ensemble empirical mode decomposition, with dispersion entropy. After that, the Gath-Geva clustering method was used to perform the diagnostic task. In [13], Ali et al. presented a feature extraction method based on empirical mode and energy entropy using an artificial neural network (ANN). Thus, the features information is extracted from the signals by statistical analysis, and then those features are classified with the help of machine-learning-based frameworks. However, most of the existing literature considers automated feature extraction procedures to augment the manual statistical analysis. Shao et al. [14] analyzed the frequency domain using Fourier transforms. After that, the Boltzmann machine was used to extract the feature information automatically. Likewise, Wang et al. [15] constructed a deep belief network (DBN)-based diagnostic model by incorporating spectrum-based features. Most researchers in this literature attempted to explore the health features of bearings by analyzing the time domain or frequency domain, making it extremely difficult to obtain invariant feature information such as the speed and load conditions varied [16]. However, numerous time-frequency based analyses have been proposed to address these issues. In [17], Sohaib et al. proposed a hybrid feature model with machine learning to automate the diagnostic process. However, for automatic feature extraction, this method is primarily focused on the analysis of one-dimensional acceleration signals [18]. Even though these methods have a bigger impact on the diagnostic framework, they can miss much of the crucial information [19]. Additionally, the above-discussed preprocessing methods are sometimes complex to build due to the necessity of proper domain expertise and may not be able to generalize the problem statement for various working conditions [20]. Fortunately, these deep learning-based diagnostic approaches improved previously prominent statistical feature-based diagnostic approaches [20]. As a result, several studies have been conducted to develop automatic feature extractor-based deep algorithms, which is a newly recognized research direction. The primary goal of these domain-dependent autonomous diagnostic systems is to create a trustworthy feature extractor that can extract different features from input data [21]. In [1], Mao et al. proposed a deep auto-encoder method based on fusing discriminant information for imbalanced data. Likewise, in [22] Xingqiu et al. introduced an optimal ensemble deep transfer network to automate the diagnosis process. Zhang et al. [23] proposed an attention mechanism to extract the features in a more reliable way by using deep learning. However, these methods have two key deficiencies: (a) They do not combine complete invariant domain knowledge with deep structures to construct a diagnostic framework that can be explained; and (b) they do not recognize numerous scenarios (e.g., fault types, bearing speed) at the same time.

To address the aforementioned shortcomings, an automatic diagnostic framework has been developed by considering knowledge from the time, frequency, and time-frequency domains, which can identify multiple health conditions (i.e., health types and bearing speed) at the same time in this paper. For preprocessing, the consideration of the time-, frequency-, and time-frequency- domains in parallel allows a multi-domain input to be built that confirmed reducing the possibility of information missing obtained from the non-stationary and non-linear vibration signals [16]. Thus, in this study, the raw time-domain signals, FFT-based frequency domain signals, and envelope analysis from the time-frequency signal are fused to generate a two-dimensional multi-domain fusion-based vibration image (*MDFVI*). Following that, a multi-task learning (MTL)-based deep architecture is developed for automatic diagnostic, which aids in the learning of many tasks simultaneously. MTL saves storage space and training time by using a shared model rather than a distinct model for each task [24]. The main objective of this special type of transfer learning (TL)-based diagnostic framework [25] is to increase the performance of all the involved tasks with the same input at the same time [25]. The contributions of this study are summarized as follows:

(1) To address variable operating conditions, a composite color image is created by fusing information from multi-domains, such as the raw time-domain signal, the spectrum of time-domain signal, and the envelope spectrum of the time-frequency analysis. This 2-D composite image, named multi-domain fusion-based vibration imaging (*MDFVI*), is highly effective to generate a unique pattern even with variable speeds and loads.
(2) The developed *MDFVI* images are further applied as inputs to the CNN-aided MTL network for automatic feature extraction and classification. The proposed network is capable of extracting features in parallel from the time-domain, the frequency-domain, and the time-frequency domain. Additionally, it is capable of predicting variable operating conditions simultaneously: (a) rotating speed and (b) fault types. As a result, multitasking capabilities for bearing fault diagnosis architecture are enabled.
(3) The proposed method is tested on two benchmark datasets from the bearing experiment. The experimental results suggested that the proposed method outperformed state-of-the-arts in both datasets.

The rest of the manuscript is organized as follows: Section 2 discusses the technical basis of FFT, envelope analysis, CNN, and MTL networks while Section 3 presents the proposed methodology, Section 4 discusses the experimental analysis, and Section 5 provides the concluding remarks of the paper.

## 2. Technical Background

This section presents the technical background of signal processing techniques, convolutional neural networks, and the basics of multi-task learning.

### 2.1. Fast-Fourier Transform (FFT)

The signals of the rolling element bearings are non-linear and non-stationary in nature [26]. For this observed phenomenon, there are hidden periodicities in the signal structure, which carry additional information. FFT is an algorithm for computing the N point discrete Fourier transform (DFT). The *N*-point DFT can be expressed as:

(1) $$X^{\left(N\right)}\left(p\right)=\sum_{n=0}^{N-1}x_{n}e^{\frac{-i2\pi pn}{N}}\;=\sum_{g}^{\frac{N}{2}-1}x_{\left(2g\right)}e^{\frac{-i2\pi pg}{\frac{N}{2}}}+e^{\frac{-i2\pi p}{N}}\sum_{h}^{\frac{N}{2}-1}x_{\left(2h+1\right)}e^{\frac{-i2\pi kh}{\frac{N}{2}}}\;=X_{0}^{\left(\frac{N}{2}\right)}\left(p\right)+e^{\frac{-i2\pi p}{N}}X_{1}{}^{\left(\frac{N}{2}\right)}\left(p\right)$$

where p=0,1,2,…,N−1 and $g,h=0,1,2,\ldots ,\frac{N}{2}-1$. In addition to that, $X_{0}^{\left(\frac{N}{2}\right)}\left(p\right)$ is the $\frac{N}{2}$ point of DFT of X(N), considered as even numbered, and $X_{1}^{\left(\frac{N}{2}\right)}\left(p\right)$ is the $\frac{N}{2}$ point of DFT of X(N), considered as odd-numbered. Moreover, both of these functions are periodic and discrete. Now, let us consider

(2) $$W_{N}=e^{\frac{-i2\pi }{N}}$$

(3) $$\mathrm{Then},\;W_{N}^{p+\frac{N}{2}}=-W_{N}^{p}$$

Here, $W_{N}^{p}$ for p=0,1,2,…,N−1 are known as the Nth root of unity. Therefore, from Equations (2), and (3), we can get,

(4) $$X^{\left(N\right)}\left(p+\frac{N}{2}\right)=X_{0}^{\frac{N}{2}}\left(p+\frac{N}{2}\right)-W_{N}^{p}X_{1}\left(p\right)$$

Here, $p=0,1,2,\ldots ,\frac{N}{2}-1$. Thus, instead of N complex multiplication, we can derive the frequency domain information from signal with $\frac{N}{2}$ multiplications. So, the computational complexity becomes O(NlogN). Therefore, by preserving the original amplitude and phase information, a fast Fourier transform (FFT) can process these vibration signals, severing them into their single sinusoidal oscillations at specific frequencies [27].

### 2.2. Envelope Analysis

When a localized fault in a rolling element bearing occurs, it interacts with another surface in the bearing each time it is loaded [28]. Vibrations are emitted as a result. Therefore, the generated periodic impulses excite many bearing resonances as well as the neighboring structure [29]. Consequently, extracting incipient information just from the frequency domain of a signal can be quite challenging. Therefore, an amplitude demodulation technique called envelope analysis is considered for extracting useful feature information from the vibration signals. To perform this analysis, it is necessary to extract the diagnostic information from the sample signal. Fortunately, the Hilbert transform demodulation technique can fabricate the analytic signal from the given sample signal to extract that information. The Hilbert transform of the real component is the imaginary factor of this analytic signal, which is a complex temporal signal. According to the following equation, the envelope e(t) of a signal x(t) is defined mathematically as the magnitude of the analytic signal.

(5) $$e\left(t\right)=\sqrt{x{\left(t\right)}^{2}+\hat{x}{\left(t\right)}^{2}}$$

In Equation (5), $\hat{x}\left(t\right)$ refers to the Hilbert transformation of the signal [28,29]. Because the bearing vibration signal is non-stationary and non-linear, Hilbert transform-based envelope analysis is used in this study to extract relevant information from the time-frequency domain.

### 2.3. Convolution Neural Network (CNN)

**Figure 1:**
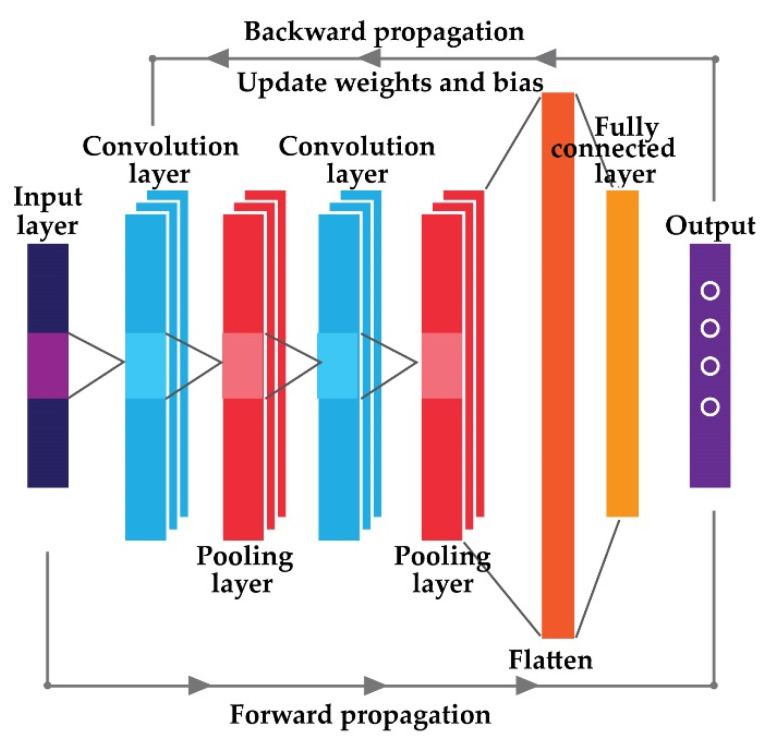
The basic design of a convolutional neural network.

A convolutional neural architecture with an input layer, several convolutions and pooling layers, multiple fully connected layers, and one output layer is a feedforward network with the benefit of automatic feature information learning and overfitting problem handling [30,31]. Furthermore, several optimization techniques, such as global pooling, dropout, and batch normalization, are frequently incorporated with the fundamental architecture of a CNN to improve the diagnostic performance [32,33,34]. Deep architectures are often trained using two main principles, as shown in Figure 1, namely (1) forward propagation and (2) backward propagation. The design usually seeks to extract spatial information from the input across the anticipated layers during the forward propagation step. During the backward propagation stage, the network attempts to alter internal parameters based on the determined objective function [35]. The main goal of these architectures is to minimize the objective function [36]. It is also worth mentioning that when it comes to deep learning-based designs, there is no hard and fast rule for establishing the optimal number of layers. The overall number of layers is determined using a train-test process that is dependent on the input data type.

#### 2.3.1. Forward Propagation

The convolution layers try to learn abstract features from the input in this step. By learning input properties with varied sizes of convolution kernels, this layer maintains the association between pixels in the input data [37]. An activation function is used in general to improve these convolved features, in addition to the added weights and bias factors [35]. The following equation can be used to describe the entire procedure:

(6) $$x_{n}^{m}=f\left(\sum_{i\in K_{n}}x_{i}^{m-1}*w_{in}^{m}+b_{n}^{m}\right)$$

In Equation (6), $x_{n}^{m}$ is the mth component of layer n, $k_{n}$ is the nth convolution region of the m−1 layer feature map, $w_{in}^{m}$ is the weight matrix, and $b_{n}^{m}$ is the added bias. After calculating the overall operation’s sum, as described in Equation (6), a non-linear activation function f called a Leaky RELU is used on it.

A pooling layer is used directly after the convolution layer to (a) remove redundancy from the retrieved features of the previous layer and (b) to reduce the number of training parameters. In this study, maxpooling is used as the pooling layer [38], which can achieve the maximum value of the convolutional output $x_{n}^{m}$ as follows:

(7) $$x_{n}^{m}=f\left(w_{n}^{m}*\max(x_{n}^{m-1})+b_{n}^{m}\right)$$

This layer is placed right after the convolution layer discussed in the previous portion. Here, the output $x_{n}^{m}$ of the convolution layer is down sampled. $w_{in}^{m}$ and $b_{n}^{m}$ are the weights and bias matrices respectively. In Equation (7), $\max\left(x_{n}^{m-1}\right)$ denotes the described maxpooling function to reduce the dimensions of the attained convoluted feature maps.

Finally, numerous convolutions and pooling layers are stacked together to boost the depth of the network design. As a result, the final completely connected layer can extract the output category from the input. Typically, numerous fully connected layers are added one after another until the final one, which changes the output matrix in the filter to a column or row [39]. The final fully connected layer can be expressed by the following Equation (8):

(8) $$y^{z}=f\left(w^{z}x^{z-1}+b^{z}\right)$$

Here, f is the activation function that produces the probabilistic output from the input in Equation (8). w and b denote the weights and bias respectively. SoftMax is used as the final activation function in this study [39].

#### 2.3.2. Backward Propagation

The objective function is determined when the forward propagation is complete to obtain the input sample’s target. This objective function is commonly referred to as a loss function. The entire procedure’s main goal is to lower the loss function between the target and actual output. The cross-entropy loss function is used in this work [35] and can be expressed as follows:

(9) $$E=\frac{1}{n}\sum_{z=1}^{n}\left[y_{z}\ln\bar{y_{z}}+(1-y_{z})\ln\left(1-\bar{y_{z}}\right)\right]$$

Here, $y_{z}$ and $\bar{y_{z}}$ are the actual target and predictive value of the zth sample, respectively. During the training procedure, the stochastic gradient descent approach is used to minimize the loss function. Due to the high computational cost of the dataset, it is not possible to train the neural network with the entire dataset at the same time [40]. Therefore, the entire dataset is divided into several smaller chunks, which are known as batches. Thus, to feed the complete dataset one-time, multiple batches are required. This process is called an epoch. To minimize the loss function by avoiding overfitting and underfitting problems, several epochs are fed to the network architecture to complete the total training process [31,40].

### 2.4. Multi-Task Learning with CNN

Multi-task learning (MTL) is a special case of transfer learning (TL) [25,41]. TL refers to the idea of transferrable knowledge. The key idea behind TL is to share the knowledge learned from a specific task with a different but relevant task. According to this principle, the main tasks in TL are generally very similar in nature, enabling the performance of the targeted tasks to be improved by sharing the trained model architecture and parameters [31,42]. Inductive learning and fine-tuned-based learning are the most suitable examples of TL [37]. Instead of sharing the model architecture separately, MTL network allows one single shared model for all the relevant tasks. Thus, MTL shares the model architecture with the trainable parameters among the relevant tasks and tries to minimize one objective function finally to generalize the model architecture [24]. Additionally, it helps to decrease the training times and reduce the storage space [43]. In this study, CNN-based MTL is used to develop the proposed diagnostic framework. This CNN-based framework simulates manifold tasks by communally learning transferable representations and task relationships [24]. The following equations express the idea of MTL:

(10) $${\left\{x_{t},y_{t}\right\}}_{t=1}^{T},where\left\{ \begin{array}{l} x_{t}=\left\{x_{1}^{t},\ldots ,x_{p}^{t}\right\}\\ y_{t}=\left\{y_{1}^{t},\ldots ,y_{p}^{t}\right\} \end{array}\right.$$

(11) $$y_{n}^{t}=f_{t}\left(x_{n}^{t}\right)$$

**Figure 2:**
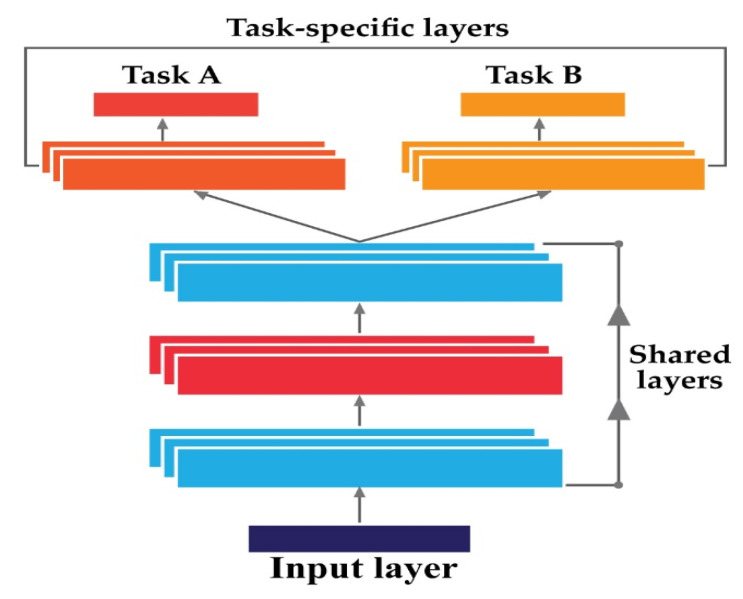
The basic concept of a multi-task learning neural network.

In Equation (10), ${\left\{x_{t},y_{t}\right\}}_{t=1}^{T}$ refers to the pair of training samples from the original task T, where $x_{t}$ refers to the individual training input, and $y_{t}$ refers to the corresponding output. p is the total number of samples present in the training dataset. The goal is to provide a diagnostic framework based on CNN for a variety of tasks $y_{n}^{t}$ for understanding and exchanging transferable factors in order to connect various tasks competently and actively. The essential principle of MTL is depicted in Figure 2 for visual comprehension. MTL-CNN is proposed in this paper for diagnostic purposes.

## 3. Proposed Methodology

**Figure 3:**
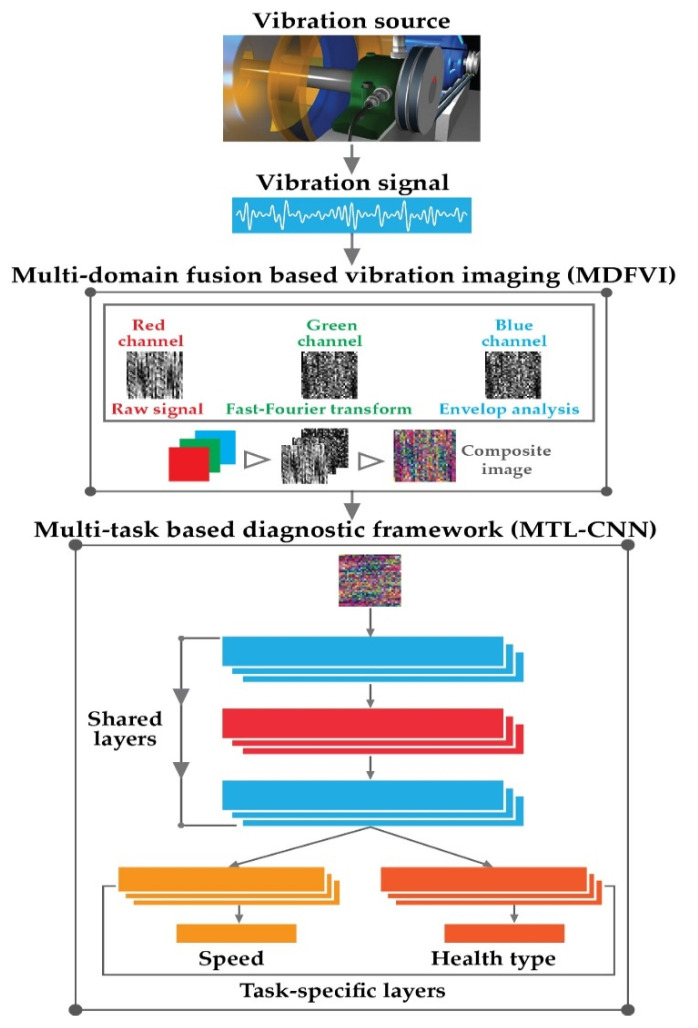
Proposed diagnostic framework.

The main purpose of this study is to determine the health statuses of rolling element bearings under changing speed settings. The suggested framework is depicted in Figure 3. As depicted in Figure 3, in the proposed framework, there are two main steps, i.e., (1) multi-domain fusion-based vibration imaging as the preprocessing step (*MDFVI*), and (2) multi-task based neural architecture (MTL-CNN) for performing the diagnostic analysis.

### 3.1. Multi-Domain Fusion Based Vibration Imaging (*MDFVI*)

Data preprocessing is a significant stage in a neural network-based diagnostic framework [44,45]. This process is challenging mainly for the following reasons: (a) the large volume of samples in the considered dataset, and (b) multiple features associated with the data. As a result, a lot of time is spent creating training samples that are highly dependent on the various operating conditions.

**Figure 4:**
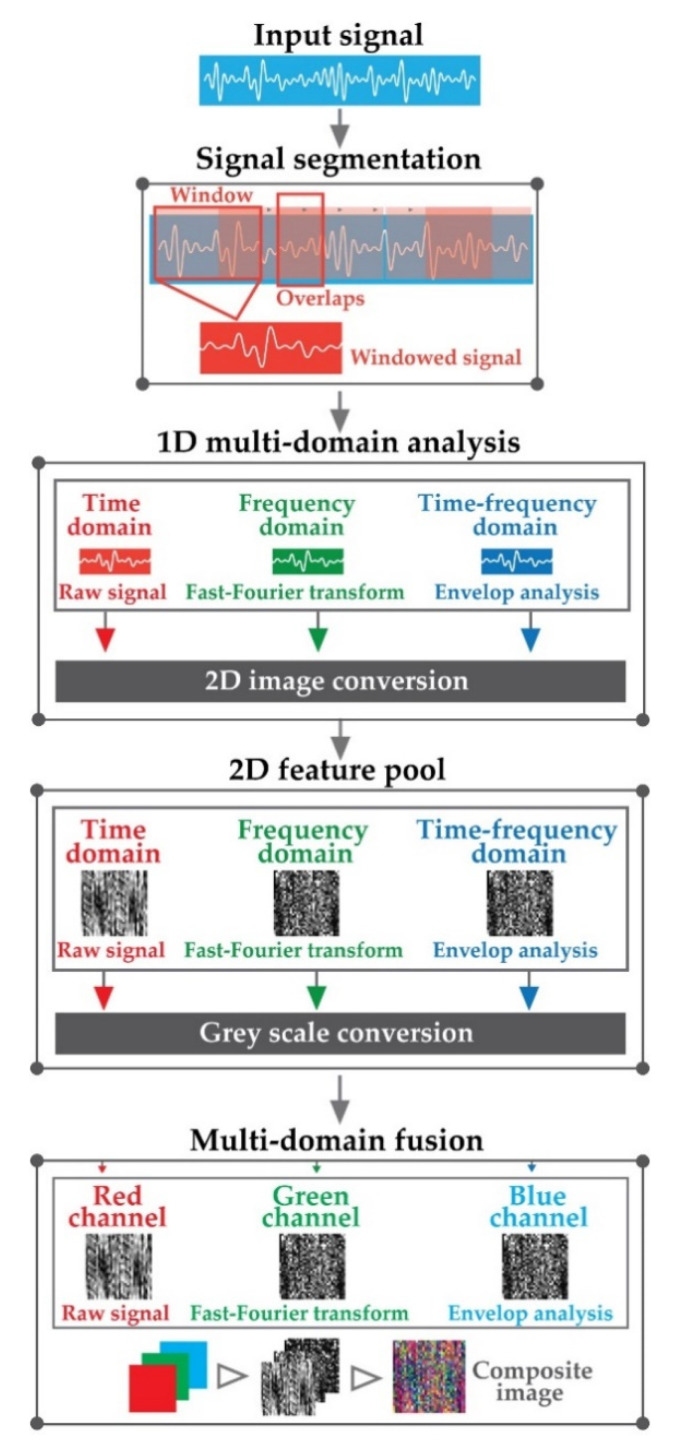
Process diagram of multi-domain fusion-based vibration imaging (*MDFVI*).

In this study, an efficient and speedy data preprocessing strategy based on increasing the characteristics of vibration signals under variable speed conditions is devised for signal-to-image conversion. The feature information is addressed in three domains in this suggested approach: (a) time domain, (b) frequency domain, and (c) time-frequency domain. Because the signal is non-stationary, neither the time domain nor the frequency domain can capture the signal’s changes [46]. Though the time-frequency domain can depict the changing of frequencies over time from non-stationary signals, it is dependent on ideal window selection procedures to find the appropriate time and frequency resolution [47]. To handle these issues, in this framework, the feature information is captured from three domains for generalizing the feature space of an individual health condition. Figure 4 illustrates the whole process. The raw vibration signals are first split into smaller portions, as seen in Figure 4 with a length 16,384 based on an overlapping window technique. Following that, (a) the time-domain information is extracted directly from the vibration signal, (b) the frequency information is extracted by FFT, and (c) the time-frequency information is extracted via envelope analysis. Later, each type of information from the considered domains (time, frequency, and time-frequency) is converted into a 2D image with a length of 128×128. Furthermore, these 2D images are converted into grayscale images. Finally, the gray-scale photos are combined to create the final *MDFVI* image, which has dimensions of 128×128×3. If 2D, time-domain grayscale image is represented as v(t), 2D frequency-domain grayscale image as $v_{f}$, and 2D grayscale envelop information to capture time-frequency information as $\hat{v}\left(t\right)$, the *MDFVI* image can be expressed as follows:

(12) $$MDFVI=v\left(t\right)+v_{f}+\hat{v}\left(t\right)$$

where, v(t), $v_{f}$, and $\hat{v}\left(t\right)$ are considered as red, green, and blue channel respectively. There are no significant reasons for these types of RGB sequences. As we have considered information from 3 domains, therefore, 3 information are considered as a color channel to form the final *MDFVI* image to get the distinguished health patterns.

### 3.2. Multi-Task Learning-Based Diagnostic Framework

**Figure 5:**
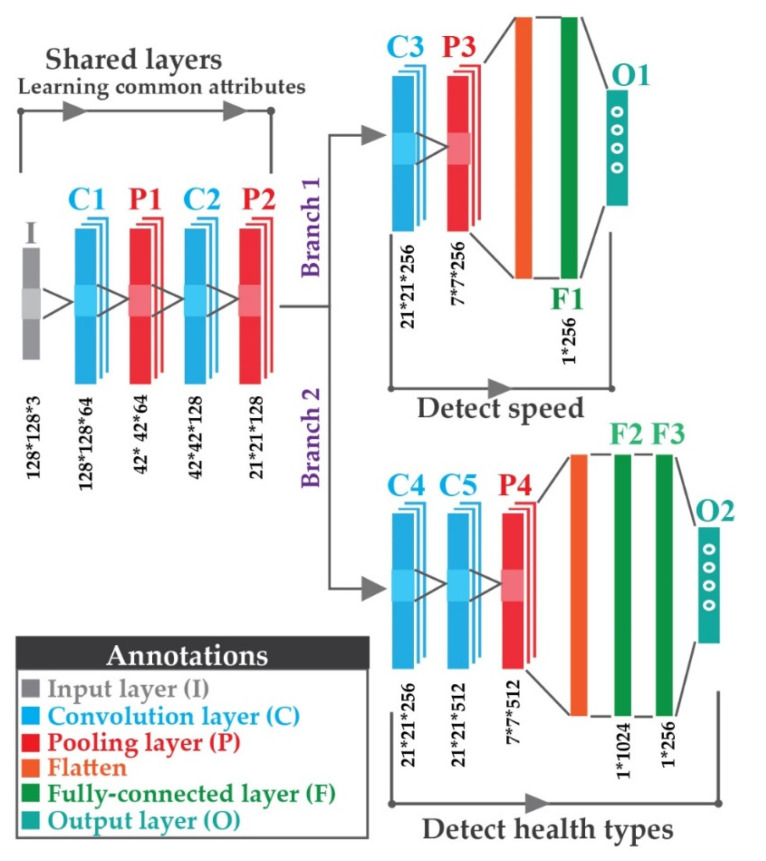
Proposed framework for CNN-based multi-task net (MTL-CNN).

For evaluating the health states of rolling element bearings under variable speed settings, the suggested MTL mechanism is based on CNN architecture. As depicted in Figure 5, the MTL-CNN architecture has two portions, (1) the common feature extractor, and (2) the task branches.

In the first portion, after the input is fed to the network, the spatial feature attributes from *MDFVI* are extracted from the subsequent layers. This portion is composed of two convolution layers and two max-pooling layers. Until this part, the network is learning the common attributes from the provided input. After that, the task branches are introduced to the proposed framework. The details of the layered architecture are depicted in Figure 5. Moreover, Leaky ReLU is considered as the activation function of the fully connected layers of both branches. On layers before the output layers for both tasks, L2 regularization of 0.05 is applied to prevent overfitting issues. There are no universally accepted guidelines for determining the overall number of layers in a model architecture. As a result, for the considered dataset, a generalized model has been constructed based on train-test methodologies and existing literature surveys [31,48].

### 3.3. Performance Evaluation Metrics

Several evaluation metrics are examined for each task for performance evaluation of the proposed framework, i.e., (1) *F*1 score (*F*1), (2) average *F*1 score (*aF*1), (3) confusion matrices [49], and (4) graph of loss functions. *F*1 and *aF*1 [50] can be obtained from the following equations:

(13) $$F1=\frac{2TP}{2TP+FN+FP}$$

(14) $$aF1=\frac{\sum F1}{Total\_classes}$$

The initials *TP*, *FP*, and *FN* in these equations stand for true positive, false positive, and false negative, respectively. Total classes indicate the total number of health types presented in the considered dataset. Furthermore, the entire loss of the model is recorded up to the defined epoch to observe the network’s bias-variance trade-off. Furthermore, the final feature space derived from the task branch is shown using t-stochastic neighbor embedding to visualize the class separation for each task (t-SNE) [51]. Subsequently, to remove the bias from the evaluation matrices, four-fold cross-validation [52] is performed to obtain the results.

## 4. Experimental Setup and Performance Analysis

The proposed framework is tested on two bearing datasets: (1) a self-designed testbed and (2) a publicly accessible repository called the Case Western Reserve University (CWRU) bearing data center [53]. Variable shaft speed and load conditions are evaluated for both datasets to validate the superiority of our suggested technique.

### 4.1. Case Study 1: Self-Designed Test Rig

#### 4.1.1. Experimental Setup and Dataset Description

**Figure 6:**
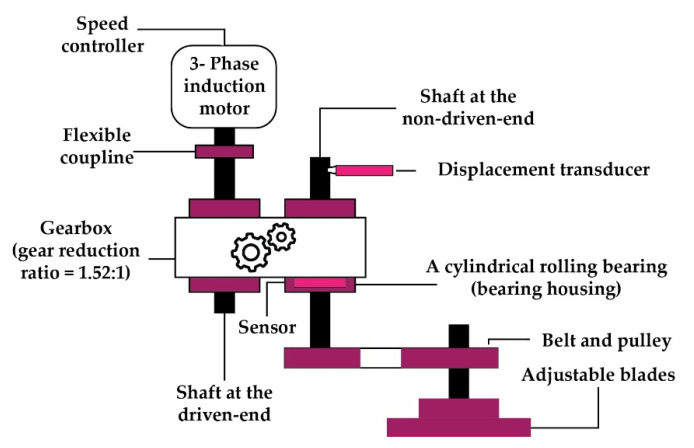
Schematic diagram of the self-designed testbed.

**Figure 7:**
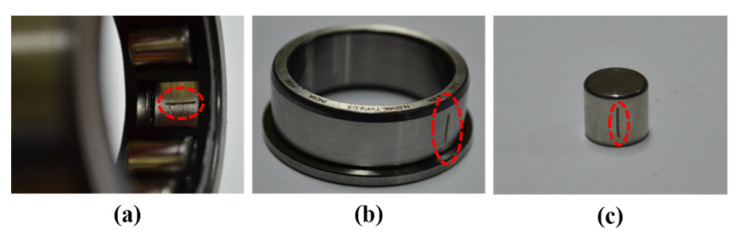
Fault types: (**a**) ORT, (**b**) IRT, and (**c**) RT.

Testing is conducted on a self-designed test rig. This rig is run at 300, 400, and 500 RPMs to obtain the vibration signal. The entire setup, as shown in Figure 6 and Figure 7, is made up of two shafts: a drive end shaft and a non-drive end shaft. To connect these two shafts, a gearbox with a reduction ratio of 1.52:1 is used. A three-phase induction motor is installed in the driving end shaft to collect data at three distinct motor speeds [54,55]. At both shaft ends of the experimental testbed, a cylindrical bearing (type FAG-NJ206-E-TVP2) is employed. A wide-band vibration sensor [56] with a sampling rate of 65536 Hz [54] is used to collect vibration signals from the non-drive end shaft. Four types of health conditions are used for conducting the experiments: normal type (NT), inner raceway type (IRT), outer raceway type (ORT), and roller type (RT). The dataset’s specifics are presented in Table 1. 

#### 4.1.2. Results and Performance Comparison

**Figure 8:**
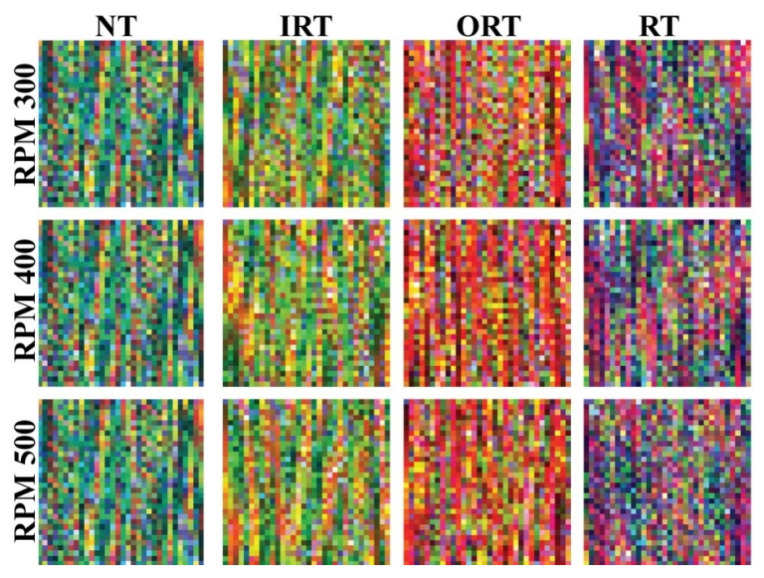
*MDFVI* representation of various health types at different speeds: NT, IRT, ORT, and RT.

The obtained *MDFVI* images from the considered four working conditions are shown in Figure 8. As can be seen in this diagram, each of the health kinds has its own set of color differences. Thus, without the necessity of any noise reduction techniques, it helps the proposed deep architecture to classify the health types. In these converted *MDFVI* images, the subtle differences are very small and difficult to identify with the bare eye. However, due to the color differences, visible distinctions can be observed. Fortunately, due to the powerful capabilities of capturing smaller changes from images, deep learning-based algorithms can help in these types of scenarios [31,57].

Additionally, from the depicted Figure 8, the consistency of color components is present in different speed conditions, which helps to establish the invariant scenarios visually. As a result, the proposed MTL-CNN is fed these *MDFVI* images for final multi-class classification. The MTL-CNN architecture’s parameters are depicted in Figure 5. The datasets considered are separated in the following ways to train and test the network.

**Figure 9:**
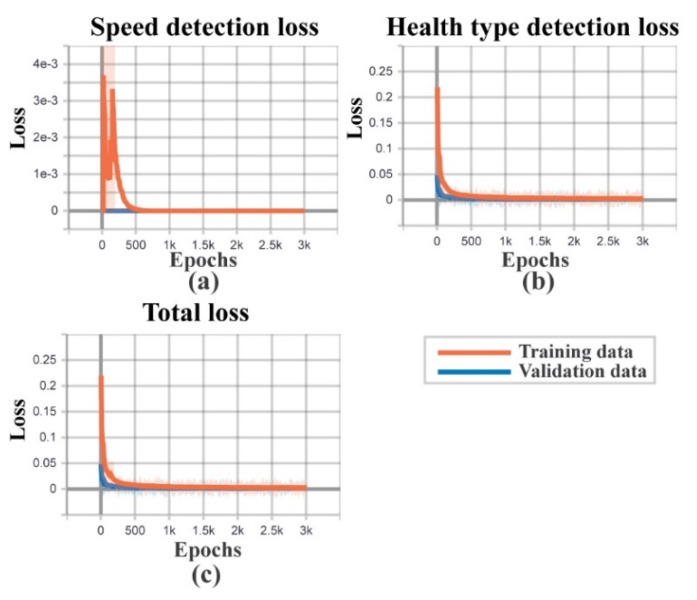
Loss functions: (**a**) training and validation loss for Task 1: speed detection, (**b**) training and validation loss for Task 2: health type identification, and (**c**) overall MTL-CNN model training and validation loss.

**Figure 10:**
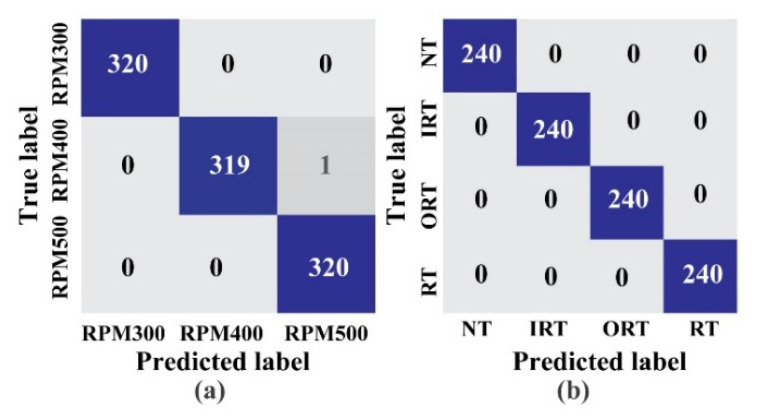
Confusion matrices: (**a**) Task 1: speed detection, and (**b**) Task 2: health type detection.

**Figure 11:**
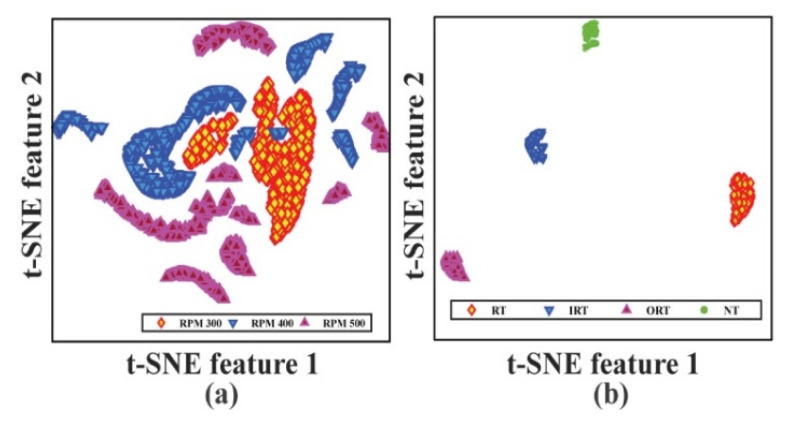
Feature space of the output layers by t-SNE: (**a**) Task 1: speed detection, and (**b**) Task 2: health type detection.

As discussed in the previous section, on each dataset, the total number of recorded signals is 800. Therefore, as listed in Table 2, a total of 1152 samples from all three datasets are used for training the network with 288 samples used for validation purposes. The remaining 960 samples are used for testing the diagnostic performance for two task branches. Furthermore, to eliminate bias, the above-mentioned data division is performed using an equal number of samples from each health class. The model is trained for 3000 epochs to validate the diagnostic performance. Besides, from Figure 9, the loss function graph can be observed for the whole model. Figure 9a highlights the loss function for speed detection, and Figure 9b shows the loss function for health type detection. Therefore, Figure 9c shows the total loss of the model. Besides, for evaluating the diagnostic performance, initially, the *F*1 and *aF*1 scores are considered from Equations (13) and (14). The diagnostic performance of the two considered work tasks are listed in Table 3. The proposed technique was 100% correct in almost every case, as shown in the table. Additionally, to make a better analysis of the obtained results, the confusion matrix (Figure 10) and the last layer of the feature space of each task are visualized by t-SNE (Figure 11). The diagnostic performance is represented in the form of actual vs. projected deviation in the confusion matrix. The proposed framework’s diagnostic performance will indeed be improved as a result of these observations.

The planned MTL-CNN is compared to different deep learning-based methodologies to determine the robustness of the proposed MTL-CNN-based diagnostic framework. These approaches draw from several sources [37,58,59], and are adapted according to the similar experimental setup as this case study. To compare the results of these methods, the af1 accuracy is employed. These techniques include the following:

(1) WC + MTL: Data are first converted into the 2D matrices of wavelet coefficient. Thus, the identification of certain frequencies is captured both in the temporal, and spatial domain [58]. Therefore, these preprocessed signals are fed into MTL-based deep architectures [59].
(2) TFI + CNN: To construct the multi-fusion input, the input is converted into many time-frequency images (TFI), which are then transferred to the MTL-CNN architecture, which is based on the proposed CNN model taken from [37].
(3) GI + CNN: The input is transformed to 2D greyscale pictures (GI), which are then fed into the MTL-CNN, which is based on the proposed CNN from [60].
(4) VMD + MTL-CNN: To generate the multifusion input, each signal is decomposed into a sequence of intrinsic mode functions using variational mode decomposition and then channel wise joined [61]. Then, using the suggested MTL-CNN architecture, those series of intrinsic mode functions are fusioned channel wise for classification.

The comparisons among these methods with the improvement details are listed in Table 4. The results show that the suggested framework (*MDFVI* + MTL-CNN) outperformed three state-of-the-art approaches, with average performance improvements of 6.58–12.51% and 6.55–13.02% for Task 1 and Task 2, respectively. In addition to that, from these results, we can claim that, for multidomain information fusion, the model can extract more meaningful information automatically. Thus, it enables the simultaneous prediction for speed and health type with a 99.99% accuracy.

The multi-domain fusion-based preprocessing approach examined in this work is confined to single sensor data. However, multiple approaches have effectively demonstrated multisensory data fusion in recent investigations. For instance, based on the belief divergence of shreds of evidence and the belief entropy, Xiao et al. [62] presented a successful fusion technique that is both practicable and effective in resolving conflicting evidence, increasing the target’s belief value to 99.05%. Similarly, Shao et al. developed a defect diagnostic technique based on multisensory fusion in [63]. For multisensory fusion, this approach proposes a stacked wavelet auto-encoder (SAE) with a Morlet wavelet. Additionally, a variable weighted assignment technique for decision fusion is devised. On the gearbox dataset, our approach displays state-of-the-art performance. These findings, however, demonstrate the critical nature of multisensory fusion for condition-based monitoring. As a result, we aim to use multisensory fusion technology for our next investigation in order to collect all relevant data from all sensor locations. Therefore, the model becomes more resilient and dependable. Additionally, several research have demonstrated effective attempts to enhance the pattern from multivariate time series. For example, Zhang et al. [64] demonstrated the use of a tri-partition state alphabet-based sequential pattern to generate a compact, understandable, and scalable pattern for multivariate time series. As a result, these findings will be beneficial for future research in order to improve the *MDFVI*’s conciseness. Furthermore, to extend the proposed MTL-CNN detection algorithm in a unsupervised one, k-means clustering techniques [65] can be useful for identifying the health cluster automatically as well.

### 4.2. Case Study 2: Case Western Reserve University Dataset

#### 4.2.1. Experimental Setup and Dataset Description

**Figure 12:**
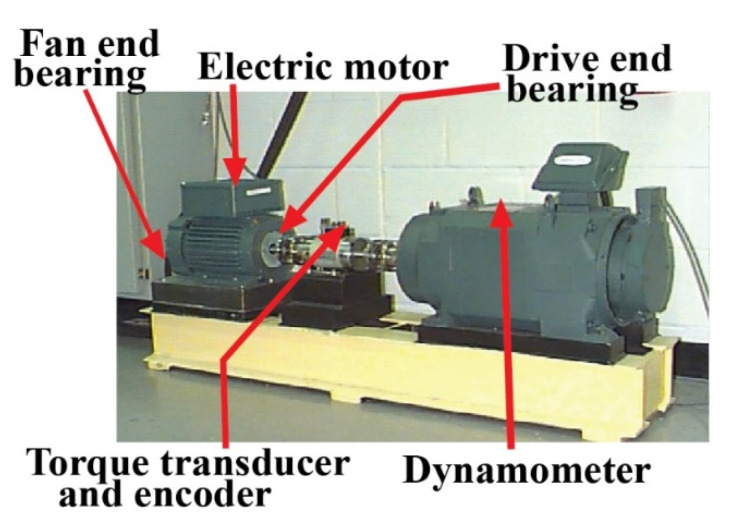
CWRU bearing testbed [66].

The vibration signals of the bearing are gathered from a public available repository, provided by Case Western Reserve University [66]. The experimental testbed is shown in Figure 12. The experimental setup consists of a 2-horsepower induction motor, a dynamometer, and a transducer, as shown in this diagram. With the help of the housing-mounted accelerometer, the desired signals are acquired by the induction motor. In addition, the dynamometer simulation considers a variety of motor loads. As a result, there is a difference in the motor shaft speeds. An electro-discharge machine is also used to manufacture the intentionally seeded defects on the driving end bearing. A sampling frequency of 12 kilohertz is used to collect the signals (kHz). As in the last case study, four types of health circumstances are used for conducting the experiments: NT, IRT, ORT, and RT. The dataset’s details are listed in Table 5.

#### 4.2.2. Verification and Performance Comparison

After the signal segmentation, to analyze the diagnostic performance from four types of health conditions, a total of 1000 signals (250 from each health type) are considered at each RPM (1797, 1772, and 1750). Then, from every sample, the *MDFVI* images are attained to feed to the proposed network. In a very similar way to the previous case study, 60% of the dataset is used for training, and the remaining 40% is used for testing. Furthermore, the MTL-CNN architecture’s parameters are kept the same as in the prior case study. The following Table 6 shows the details of the data split. According to the previous explanation, the model is also trained for 3000 epochs with four-fold cross-validation.

For calculating the diagnostic performance, the *F*1 and *aF*1 scores are calculated from Equations (13) and (14). The analytical performances are given in Table 7. 

From these analyses, it can be ensured and validated that the proposed approach can provide a reasonable state-of-the-art diagnostic performance. Furthermore, the achieved 100% accuracy in the entire considered scenario indicates the generalization ability of the proposed approach. Similarly, as in the previous case study, to establish the generalization ability of this MTL-CNN-based diagnostic framework, the designed framework is compared with these previously mentioned approaches, i.e., (1) WC + MTL [59], (2) TFI + CNN [37], and (3) GI + CNN [60]. For these diagnostic frameworks, the preprocessing details and the parameters are kept similar to those used in the previous case study. The details of the comparisons are listed in Table 8.

**Figure 13:**
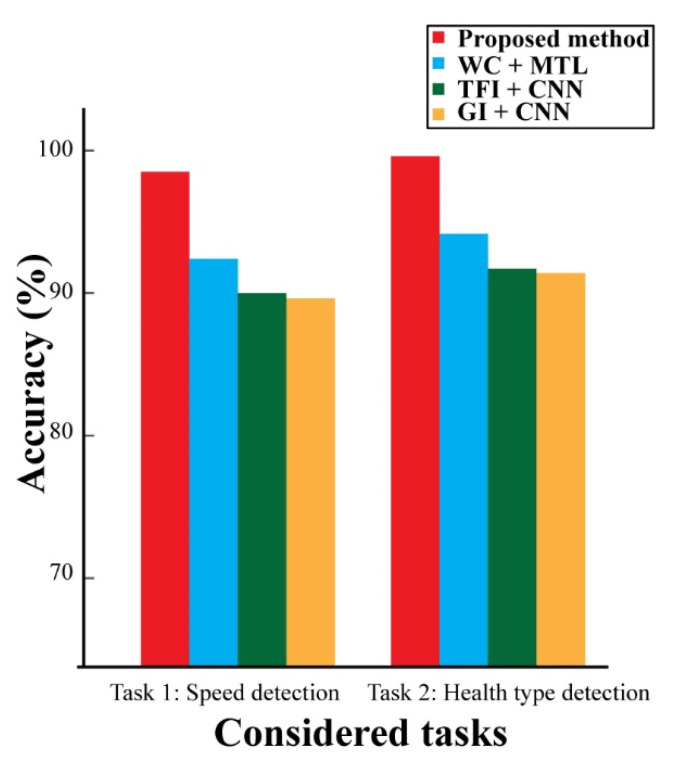
The effects of noisy data on the diagnostic performance of various methods.

For the CWRU dataset, the suggested framework (*MDFVI* + MTL-CNN) beat three state-of-the-art approaches, delivering an average performance enhancement of 1.21–6.59% and 1.87–6.45% for Task 1 and Task 2, respectively. Furthermore, the effects of noise on diagnostic performance have been examined for easy replication using this freely available dataset. Gaussian white noise with a signal-to-noise ratio (SNR) of 6 dB is introduced to the testing samples to replicate data with additional background noise. Before being tested on the simulated noisy data, all similar techniques, including the proposed one, are trained on the original preprocessed input data. Figure 13 shows the diagnostic results. Due to the noisy dataset, the diagnostic performances of all the evaluated approaches have gone off, according to this analysis. However, the proposed model outperforms the alternatives.

## 5. Conclusions

This study demonstrated an autonomous diagnostic system that combines signal-to-image translation techniques for multi-domain information with convolutional neural network-assisted multitask learning. One of primary objectives of this study is to manage variable operating conditions such as varying loads and speeds. As a result, to accommodate changing operating conditions, a composite color image is created by fusing data from many domains, including the raw time-domain signal, the time-domain signal’s spectrum, and the time-frequency analysis’s envelope spectrum. This two-dimensional composite picture technique, called multi-domain fusion-based vibration imaging (*MDFVI*), is particularly effective at creating a unique pattern independent of speed or load. Following that, these *MDFVI* images are fed into the proposed MTL-based CNN architecture, which is capable of accurately detecting flaws in changing speed and health conditions concurrently. However, the proposed preprocessing method studied in this work is currently limited to data from a single sensor. Additionally, the proposed framework is now constrained to the fixed resolution of *MDFVI*. As a result, we want to conduct our next experiment using multisensory fusion technology in order to capture all essential data from all sensor locations. Furthermore, future work will incorporate an adaptive time, frequency, and time-frequency resolution when constructing a robust *MDFVI* as an input. As a result, the model becomes more robust and reliable.

## Figures and Tables

**Table 1:** Details about the working environment for case study 1.

	Health Type	Shaft Speed (rpm)	Crack Size
			Length (mm)
**Dataset 1**	NT	300	-
	IRT		6
	ORT		6
	RT		6
**Dataset 2**	NT	400	-
	IRT		6
	ORT		6
	RT		6
**Dataset 3**	NT	500	-
	IRT		6
	ORT		6
	RT		6

**Table 2:** The train, test, and validation dataset ratios.

Dataset	Train (60%)		Test (40%)	Total Samples	Sample/Health Type
	Training (80%)	Validation (20%)			
**1**	384	96	320	800	200
**2**	384	96	320	800	200
**3**	384	96	320	800	200
**Total**	**1152**	**288**	**960**		

**Table 3:** Diagnostic performance of the dataset.

Tasks	Conditions	*F*1 (%)	*aF*1 (%)
Task 1: Speed detection	300 RPM	100	99.99
	400 RPM	99.99	
	500 RPM	100	
Task 2: Health type detection	NT	100	100
	IRT	100	
	ORT	100	
	RT	100	

**Table 4:** Comparison analysis for case study 1.

Methods	Tasks	*aF*1 (%)	Improvement (Proposed − Current)
WC + MTL	Task 1	91.21	99.99 − 91.21 = **8.78**
	Task 2	93.45	100 − 93.45 = **6.55**
TFI + CNN	Task 1	93.41	99.99 − 93.41 = **6.58**
	Task 2	93.95	100 − 93.95 = **6.05**
GI + CNN	Task 1	87.48	99.99 − 87.48 = **12.51**
	Task 2	86.92	100 − 86.92 = **13.08**
VMD + MTL-CNN	Task 1	81.38	100 − 81.38 = **18.62**
	Task 2	80.52	100 − 80.52 = **19.48**
Proposed	Task 1	99.99	-
	Task 2	100	-

**Table 5:** Dataset for case study 2.

	Health Type	RPM	Load	Crack Size
				Length (Inches)
**Dataset 1**	NT	1797	0	-
	IRT		0	0.007
	ORT		0	0.007
	BT		0	0.007
**Dataset 2**	NT	1772	1	-
	IRT		1	0.007
	ORT		1	0.007
	BT		1	0.007
**Dataset 3**	NT	1750	2	-
	IRT		2	0.007
	ORT		2	0.007
	BT		2	0.007

**Table 6:** The training, testing, and validation dataset ratios.

Dataset	Training (60%)		Testing (40%)	Total Samples	Sample/Health Type
	Training (80%)	Validation (20%)			
**1**	480	120	400	1000	250
**2**	480	120	400	1000	250
**3**	480	120	400	1000	250
**Total**	**1440**	**360**	**1200**		

**Table 7:** Diagnostic performance of the CWRU dataset.

Tasks	Conditions	*F*1 (%)	*aF*1 (%)
Task 1: Speed detection	1797 RPM	100	100
	1772 RPM	100	
	1750 RPM	100	
Task 2: Health type detection	NT	100	100
	IRT	100	
	ORT	100	
	RT	100	

**Table 8:** Comparison of the diagnostic performance for case study 2.

Methods	Tasks	*aF*1 (%)	Improvement (Proposed − Reference Model)
WC + MTL	Task 1	96.21	100 − 96.21 = **3.79**
	Task 2	97.43	100 − 97.43 = **2.57**
TFI + CNN	Task 1	98.79	100 − 98.79 = **1.21**
	Task 2	98.13	100 − 93.13 = **1.87**
GI + CNN	Task 1	93.41	100 − 93.41 = **6.59**
	Task 2	93.55	100 − 93.55 = **6.45**
Proposed	Task 1	100	-
	Task 2	100	-

## Data Availability

The data from case study 2 is publicly available.

## References

[1] Mao W., Feng W., Liu Y., Zhang D., Liang X. (2021). A new deep auto-encoder method with fusing discriminant information for bearing fault diagnosis. Mech. Syst. Signal Process..

[2] Chen X., Zhang B., Gao D. (2021). Bearing fault diagnosis base on multi-scale CNN and LSTM model. J. Intell. Manuf..

[3] Yan X., Liu Y., Jia M., Zhu Y. (2019). A multi-stage hybrid fault diagnosis approach for rolling element bearing under various working conditions. IEEE Access.

[4] Hasan M.J., Kim J., Kim C.H., Kim J.-M. (2020). Health State Classification of a Spherical Tank Using a Hybrid Bag of Features and K-Nearest Neighbor. Appl. Sci..

[5] Mao W., Chen J., Liang X., Zhang X. (2019). A new online detection approach for rolling bearing incipient fault via self-adaptive deep feature matching. IEEE Trans. Instrum. Meas..

[6] Rai A., Kim J.-M. (2020). A novel health indicator based on the Lyapunov exponent, a probabilistic self-organizing map, and the Gini-Simpson index for calculating the RUL of bearings. Measurement.

[7] Hasan M.J., Sohaib M., Kim J.-M. (2020). A Multitask-Aided Transfer Learning-Based Diagnostic Framework for Bearings under Inconsistent Working Conditions. Sensors.

[8] Qu J., Zhang Z., Gong T. (2016). A novel intelligent method for mechanical fault diagnosis based on dual-tree complex wavelet packet transform and multiple classifier fusion. Neurocomputing.

[9] Chen G., Liu F., Huang W. (2017). Sparse discriminant manifold projections for bearing fault diagnosis. J. Sound Vib..

[10] Zheng H., Wang R., Yang Y., Li Y., Xu M. (2019). Intelligent fault identification based on multisource domain generalization towards actual diagnosis scenario. IEEE Trans. Ind. Electron..

[11] Oh H., Jung J.H., Jeon B.C., Youn B.D. (2017). Scalable and unsupervised feature engineering using vibration-imaging and deep learning for rotor system diagnosis. IEEE Trans. Ind. Electron..

[12] Zhang X., Zhang M., Wan S., He Y., Wang X. (2021). A bearing fault diagnosis method based on multiscale dispersion entropy and GG clustering. Measurement.

[13] Ali J.B., Fnaiech N., Saidi L., Chebel-Morello B., Fnaiech F. (2015). Application of empirical mode decomposition and artificial neural network for automatic bearing fault diagnosis based on vibration signals. Appl. Acoust..

[14] Shao S.-Y., Sun W.-J., Yan R.-Q., Wang P., Gao R.X. (2017). A deep learning approach for fault diagnosis of induction motors in manufacturing. Chin. J. Mech. Eng..

[15] Wang X., Huang J., Ren G., Wang D. (2017). A hydraulic fault diagnosis method based on sliding-window spectrum feature and deep belief network. J. Vibroeng..

[16] Yan X., Jia M. (2018). A novel optimized SVM classification algorithm with multi-domain feature and its application to fault diagnosis of rolling bearing. Neurocomputing.

[17] Sohaib M., Kim C.-H., Kim J.-M. (2017). A Hybrid Feature Model and Deep-Learning-Based Bearing Fault Diagnosis. Sensors.

[18] Wang H., Li S., Song L., Cui L., Wang P. (2019). An enhanced intelligent diagnosis method based on multi-sensor image fusion via improved deep learning network. IEEE Trans. Instrum. Meas..

[19] Huang R., Liao Y., Zhang S., Li W. (2018). Deep decoupling convolutional neural network for intelligent compound fault diagnosis. IEEE Access.

[20] Zhang W., Peng G., Li C., Chen Y., Zhang Z. (2017). A new deep learning model for fault diagnosis with good anti-noise and domain adaptation ability on raw vibration signals. Sensors.

[21] Kang M., Islam M.R., Kim J., Kim J.M., Pecht M. (2016). A Hybrid Feature Selection Scheme for Reducing Diagnostic Performance Deterioration Caused by Outliers in Data-Driven Diagnostics. IEEE Trans. Ind. Electron..

[22] Ince T., Kiranyaz S., Eren L., Askar M., Gabbouj M. (2016). Real-Time Motor Fault Detection by 1-D Convolutional Neural Networks. IEEE Trans. Ind. Electron..

[23] Zhang T., Liu S., Wei Y., Zhang H. (2021). A novel feature adaptive extraction method based on deep learning for bearing fault diagnosis. Measurement.

[24] Dobrescu A., Giuffrida M.V., Tsaftaris S.A. (2020). Doing More With Less: A Multitask Deep Learning Approach in Plant Phenotyping. Front. Plant Sci..

[25] Pan S.J., Yang Q. (2009). A survey on transfer learning. IEEE Trans. Knowl. Data Eng..

[26] Randall R.B., Antoni J. (2011). Rolling element bearing diagnostics-A tutorial. Mech. Syst. Signal Process..

[27] Pang B., Tang G., Tian T., Zhou C. (2018). Rolling Bearing Fault Diagnosis Based on an Improved HTT Transform. Sensors.

[28] Sadoughi M., Hu C. (2019). Physics-based convolutional neural network for fault diagnosis of rolling element bearings. IEEE Sens. J..

[29] Howard I. (1994). A Review of Rolling Element Bearing Vibration, Detection, Diagnosis and Prognosis.

[30] Lecun Y., Bengio Y., Hinton G. (2015). Deep learning. Nature.

[31] Hasan M.J., Islam M.M.M., Kim J.M. (2019). Acoustic spectral imaging and transfer learning for reliable bearing fault diagnosis under variable speed conditions. Meas. J. Int. Meas. Confed..

[32] Srivastava N., Hinton G., Krizhevsky A., Sutskever I., Salakhutdinov R. (2014). Dropout: A simple way to prevent neural networks from overfitting. J. Mach. Learn. Res..

[33] Ioffe S., Szegedy C. (2015). Batch normalization: Accelerating deep network training by reducing internal covariate shift. arXiv.

[34] Dahl G.E., Sainath T.N., Hinton G.E. (2013). Improving deep neural networks for LVCSR using rectified linear units and dropout. Proceedings of the 2013 IEEE International Conference on Acoustics, Speech and Signal Processing.

[35] Wang H., Xu J., Yan R., Gao R.X. (2019). A New Intelligent Bearing Fault Diagnosis Method Using SDP Representation and SE-CNN. IEEE Trans. Instrum. Meas..

[36] Zhao M., Kang M., Tang B., Pecht M. (2019). Multiple Wavelet Coefficients Fusion in Deep Residual Networks for Fault Diagnosis. IEEE Trans. Ind. Electron..

[37] Wang J., Mo Z., Zhang H., Miao Q. (2019). A deep learning method for bearing fault diagnosis based on time-frequency image. IEEE Access.

[38] Jing L., Zhao M., Li P., Xu X. (2017). A convolutional neural network based feature learning and fault diagnosis method for the condition monitoring of gearbox. Measurement.

[39] Ma J., Wu F., Zhu J., Xu D., Kong D. (2017). A pre-trained convolutional neural network based method for thyroid nodule diagnosis. Ultrasonics.

[40] Brownlee J. (2018). What is the Difference Between a Batch and an Epoch in a Neural Network?. Mach. Learn. Mastery.

[41] Ruder S. (2017). An overview of multi-task learning in deep neural networks. arXiv.

[42] Hasan M.J., Kim J.-M. (2018). Bearing Fault Diagnosis under Variable Rotational Speeds Using Stockwell Transform-Based Vibration Imaging and Transfer Learning. Appl. Sci..

[43] Long M., Cao Z., Wang J., Philip S.Y. Learning multiple tasks with multilinear relationship networks. Proceedings of the Advances in Neural Information Processing Systems.

[44] Hoang D.-T., Kang H.-J. (2019). Rolling element bearing fault diagnosis using convolutional neural network and vibration image. Cogn. Syst. Res..

[45] Hasan M.J., Kim J.-M. Deep Convolutional Neural Network with 2D Spectral Energy Maps for Fault Diagnosis of Gearboxes under Variable Speed. Proceedings of the Mediterranean Conference on Pattern Recognition and Artificial Intelligence.

[46] Tao H., Wang P., Chen Y., Stojanovic V., Yang H. (2020). An unsupervised fault diagnosis method for rolling bearing using STFT and generative neural networks. J. Frankl. Inst..

[47] Yin Q., Shen L., Lu M., Wang X., Liu Z. (2013). Selection of optimal window length using STFT for quantitative SNR analysis of LFM signal. J. Syst. Eng. Electron..

[48] Cao P., Zhang S., Tang J. (2018). Preprocessing-Free Gear Fault Diagnosis Using Small Datasets with Deep Convolutional Neural Network-Based Transfer Learning. IEEE Access.

[49] Luque A., Carrasco A., Martín A., de las Heras A. (2019). The impact of class imbalance in classification performance metrics based on the binary confusion matrix. Pattern Recognit..

[50] Goutte C., Gaussier E. (2005). A probabilistic interpretation of precision, recall and F-score, with implication for evaluation. Proceedings of the European Conference on Information Retrieval.

[51] van der Maaten L., Hinton G. (2008). Visualizing data using t-SNE. J. Mach. Learn. Res..

[52] Browne M.W. (2000). Cross-validation methods. J. Math. Psychol..

[53] Case Western Reserve University (2017). Case Western Bearing Data Center. https://engineering.case.edu/bearingdatacenter.

[54] Sohaib M., Kim J.-M. (2019). Fault diagnosis of rotary machine bearings under inconsistent working conditions. IEEE Trans. Instrum. Meas..

[55] Hasan M.J., Sohaib M., Kim J.-M. (2021). An Explainable AI-Based Fault Diagnosis Model for Bearings. Sensors.

[56] Piezotronic P. Sensor Details. http://www.pcb.com/contentstore/mktgContent/IMI_Downloads/IM%0AI-RouteBased_LowRes.pdf.

[57] Amar M., Gondal I., Wilson C. (2015). Vibration spectrum imaging: A novel bearing fault classification approach. IEEE Trans. Ind. Electron..

[58] Hoang D.T., Kang H.J. (2019). A Motor Current Signal-Based Bearing Fault Diagnosis Using Deep Learning and Information Fusion. IEEE Trans. Instrum. Meas..

[59] Guo S., Zhang B., Yang T., Lyu D., Gao W. (2019). Multitask Convolutional Neural Network with Information Fusion for Bearing Fault Diagnosis and Localization. IEEE Trans. Ind. Electron..

[60] Pucciarelli G. (2017). Wavelet analysis in volcanology: The case of phlegrean fields. J. Environ. Sci. Eng. A.

[61] Cui H., Guan Y., Chen H. (2021). Rolling element fault diagnosis based on VMD and sensitivity MCKD. IEEE Access.

[62] Xiao F. (2019). Multi-sensor data fusion based on the belief divergence measure of evidences and the belief entropy. Inf. Fusion.

[63] Shao H., Lin J., Zhang L., Galar D., Kumar U. (2021). A novel approach of multisensory fusion to collaborative fault diagnosis in maintenance. Inf. Fusion.

[64] Zhang Z.-H., Min F., Chen G.-S., Shen S.-P., Wen Z.-C., Zhou X.-B. (2021). Tri-Partition State Alphabet-Based Sequential Pattern for Multivariate Time Series. Cognit. Comput..

[65] Ran X., Zhou X., Lei M., Tepsan W., Deng W. (2021). A novel k-means clustering algorithm with a noise algorithm for capturing urban hotspots. Appl. Sci..

[66] Case Western Reserve University Bearing Data Center Website. http://csegroups.case.edu/bearingdatacenter/pages/download-data-file.

